# Molecular identification of *Austrodiplostomum* sp., an eye parasite among farmed tambaqui in Amazonia

**DOI:** 10.1590/1678-4685-GMB-2021-0345

**Published:** 2023-01-30

**Authors:** Eduardo Freitas de Farias, Hallana Cristina Menezes da Silva, Ana Paula Costa de Carvalho, Rodrigo de Melo Martins, Andrea Belem-Costa, Wallice Paxiúba Duncan, Ana Lúcia Silva Gomes, Daniele Aparecida Matoso

**Affiliations:** 1 Instituto Nacional de Pesquisas da Amazônia, Programa de Pós-Graduação em Genética, Conservação e Biologia Evolutiva, Manaus, AM, Brazil.; 2 Universidade Federal do Amazonas, Instituto de Ciências Biológicas, Departamento de Parasitologia, Laboratório de Imunologia de Animais Aquáticos. Manaus, AM, Brazil.; 3 Universidade Federal do Amazonas, Instituto de Ciências Biológicas, Departamento de Morfologia, Laboratório de Morfologia Funcional, Manaus, AM, Brazil.; 4 Universidade Federal do Amazonas, Instituto de Ciências Biológicas, Departamento de Parasitologia, Laboratório de Parasitologia de Animais Aquáticos, Manaus, AM, Brazil.; 5 Universidade Federal do Amazonas, Instituto de Ciências Biológicas, Departamento de Genética, Laboratório de Biotecnologia e Citogenômica Animal, Manaus, AM, Brazil.

**Keywords:** Metacercariae, parasitism, Colossoma macropomum

## Abstract

*Austrodiplostomum* Szidat & Nani, 1951 is a genus of parasitic digenetic trematodes widely distributed in the Neotropical region. Infects a wide variety of species, families and requests for freshwater fish. We identify samples of *Austrodiplostomum* sp, based on metacercariae isolates from eyes of tambaqui (*Colossoma macropomum*), a fish of high commercial importance in Brazil, and widely consumed by the population of the northern region. The sequences obtained clustered with *A. compactum*. This is the first report of the occurrence of diplostomids in farmed tambaqui in Amazonia.

## Introduction

Parasites use other organisms as support for food, shelter, and transportation. Their developmental stages may involve more than one host species in a number of higher taxa. *Austrodiplostomum* Szidat and Nani 1951 is a genus of parasitic trematodes in the family Diplostomidae (order Diplostomida). The genus is widely distributed in the Neotropical region and has molluscs and fish as intermediate hosts (Monteiro et al., 2016). On entering the host bloodstream, these parasites eventually reach the eyes and transform into metacercariae. During this life stage, they can compromise host development by additionally infecting a number of organs, including gills, muscle, swimming bladder and brain. Freshwater diplostomides colonize fish of different species, families and orders (Martins et al., 1999; Ramos et al., 2013). Taxonomic studies of parasitic organisms in fish help define which type of parasite is present in a given host, allowing for searches to be made for mitigating measures and the development of good management practices that will prevent infestation proliferation (Florindo et al., 2017). Approaches to the study and definition of groups of fish parasites based on purely morphological analysis may encounter difficulties because the identification of larval stages, in many cases, can only be clarified with molecular analyses (Moszczynska et al., 2009). The use of molecular markers in studies to study genetic variability and provide species-specific diagnosis for different forms of parasitic organisms provides an alternative approach to those more traditional studies using only morphological and etiological characters (Sperling et al., 1994; Otranto and Stevens, 2002).

The information generated in this study will provide extra input and can be used as primary data to combat and control of these pathogenic organisms, by improvement of zootechnical methods for this commercially important fish species, widely cultivated in the Amazon region. 

## Material and Methods

### Sample collection and morphological analysis

The fish were collected at the Balbina Fish Farm Station, the largest fingerling producing unit in the State of Amazonas. It is located in the coordinates (2˚4'5'' S; 60˚31'58'' W) in a hydroelectric area in the Presidente Figueiredo city. Metacercariae of the genus *Austrodiplostomum*, parasitizing *Colossoma macropomum* (tambaqui), were carefully removed from the eyes of infected fish, after anaesthetized by benzocaine. Some of the specimens (6), were preserved in alcohol while others (7) were preserved in formalin (36%), for, respectively, analysis with scanning electron (SEM) and optical microscopy. A series of 12 metric dimensions were taken from 13 metacercariae specimens. Morphological characterization followed the methods of Santos et al. (2002), Kohn et al. (1995), Novaes et al. (2006) and Albuquerque et al. (2017). Samples were deposited in the Helminthological Collection of the Amazon National Research Institute (INPA). All procedures described were approved by the UFAM Ethics Committee for Animal Experimentation, under permit number 002/14-CEUA/UFAM.

### DNA sequence analysis

Total metacercarian DNA was extracted using a PureLink™ Genomic DNA Mini Kit, Invitrogen (Thermo Fisher Scientific), and following the manufacturer’s instructions. This was followed by a PCR treatment using the GoTaq® Hot Start Polymerase kit, (Promega). Primers were as described by Moszczynska et al. (2009) for the amplification of the Cytochrome Oxidase Subunit I (COI) region of the mitochondrial DNA of metacercaria. PCR reactions had a final volume of 20 µL, with: 10.8 µL of ultra-pure water, 0.2 µL of dNTP, 1.2 µL of MgCl2, 0.8 µL of forward primer and 10 mM of reverse primer, 0.2 µL of GoTaq® Hot Start Polymerase and 2 µL of 50 ng DNA. In the thermal cycler, samples were submitted to the following conditions: initial denaturation at 94 °C for 5 min, followed by 35 denaturation cycles at 94 °C for 30 s, annealing at 50 °C for 30 s and extension at 72 °C for 1 min, followed by a final extension at 72 °C for 10 min. To cross-check the PCR technique, a 1.5% agarose gel electrophoresis was performed, immersed in TBE1X, with 80 V electric current, for 40 min. The gel was then observed under UV light.

Following the positive PCR reaction result, samples were purified using the *ExoSap* protocol. Sequencing reactions used the BigDye™ kit Terminator v3.1 Cycle Sequencing, Applied Biosystems (Thermo Fisher Scientific), following the manufacturer’s instructions. Samples were purified using the NG purification protocol. Sequencing was performed in an automatic sequencer (Sanger ABI 3130, Applied Biosystems). For molecular analyses of Maximum Likelihood (ML) and Bayesian Inference (BI) were analysed 34 sequences (1 of *D. huronense* HM064670, 1 of *D. spathaceum* KR271467, 8 of *A. compactum* MH378944-MH378951, 4 of *Austrodiplostomum* sp2 MH378931-MH378934, 3 of *A*. sp1 MH378928-MH378930, 3 of *A. mordax* MH378896-MH378898, 2 *A. ostrowskiae* KT728794-KT728795, 12 new sequences of *A. compactum* MT271131- MT271142). Partial sequences containing 416 bases corresponding to the COI gene, were edited and aligned using the BioEdit editor Clustal W program (Hall, 1999). We used the PAUP* program (Swofforf, 1998), for obtain the best evolutionary model by using Modeltest 3.7 (Posada and Crandall, 1998) under the Akaike Information Criterion (AIC). The best model was GTR+G+I, which was used for Maximum Likelihood (ML) analyses. Bootstrap Phylogeny test with 1050 replications and heuristic search to assess branch support was performed in ML analyses using the MEGA 10.1.7 program (Kumar et al., 2018). Species relationships within *Austrodiplostomum* were assessed using Bayesian inference (BI). BI analysis was carried out with MrBayes v. 3.2.7 (Ronquist et al., 2012) on the using Markov chain Monte Carlo searches on two simultaneous runs of four chains for 10^6^ generations, sampling trees every 10^3^ generations. The burn-in was set for the 25% of the trees sampled. A consensus topology and nodal support estimated as posterior probability values (Huelsenbeck et al., 2001) were calculated from the remaining trees. Phylogenetic trees were visualised and finalised in FigTree v. 1.4.4 (http://tree.bio.ed.ac.uk/software/figtree/). 

## Results and Discussion

**Figure 1 -:**
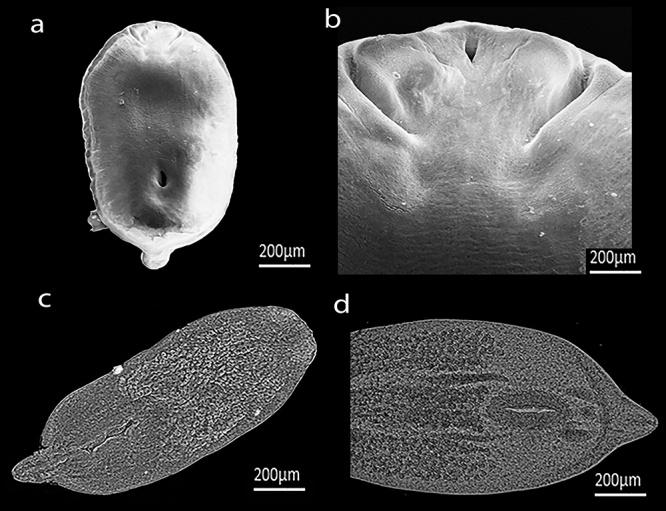
*Austrodiplostomum compactum* metacercariae scanning electron micrograph. (a,c) Whole specimen, (b) anterior region evidencing oral suction cup and pseudoventer, (d) tribocytic organ.

Metacercariae collected from *C. macropomum* vitreous humor were measured and characterized morphologically as follows: body foliaceous, bisegmented, anterior region with ventral portion with a slight depression; integument papillaceous; subterminal oral suction cup small, flanked by two well-developed pseudoventae; tribocytic organ present, with a large number of glandular cells spread throughout the anterior region; posterior region consisting of a reduced tapering process (Figure 1). Morphometric data recorded from the metacercariae samples appears in Tables 1 and 2. 

**Table 1 -:** Morphometric data of *Austrodiplostomum compactum* metacercariae, (fish from Brazil), with respective localities and hosts and sample size.

Author/Locality/Host/sample size	Body		Oral sucker		Pharynx		Tribocytic organ	
	length	width	length	width	length	width	length	width
Khon *et al*., 1995 Hidreletrica de Itaipu - PR *Plagioscion squamosissimus* N = (14)	2170 (1470-2740)	970 (600-1180)	77 (41-97)	79 (56-116)	83 (64-94)	60 (45-79)	507 (326-650)	370 (251-500)
Santos et al., 2002 Paraná River - SP *Plagioscion squamosissimus* N = (20)	1434 (880-1840)	611 (400-792)	65 (44-90)	52 (40-64)	62 (50-64)	49 (40-60)	285 (200-600)	182 (160-232)
Santos et al., 2002 Paraná River - SP *Cichla ocellaris* N = (20)	1462 (069-2480)	711 (560-960)	68 (40-98)	56 (30-98)	68 (44-98)	52 (38-78)	308 (200-496)	180 (120-320)
Paes et al., 2003 Tiête River - SP *Plagioscion squamosissimus* N = (21)	1911 (1301-2386)	678 (482-854)	71 (51-87)	73 (51-92)	69 (49-84)	58 (45-74)	401 (205-554)	246 (127-347)
Novaes et al., 2006 Reservatório Barra Bonita - SP *Geophagus brasiliensis* N = (05)	1880 (1584-1947)	642 (537-706)	59 (45-83)	68 (54-77)	61 (53-73)	56 (50-64)	428 (422-434)	258 (220-319)
Zica et al., 2009 Reservatório de Chavantes - SP *Hypostomus regani* N = (10)	1988 (1570-2281)	756 (543-864)	91 (69-102)	84 (75-99)	73 (57-85)	64 (57-80)	373 (287-414)	243 (178-310)
Lepera *et al*., 2017 Tietê River - SP *Plagioscion squamosissimus* N = 50	1789 (1450-2166)	683 (566-902)	72 (57-86)	76 (49-97)	80 (61-99)	58 (41-75)	405 (302-492)	256 (193-319)

**Table 2 -:** Morphometric data of *Austrodiplostomum compactum* metacercariae (fish from the Amazon basin), with measurements, localities, hosts and sample size.

Author/Locality/Host/sample size	Body		Oral sucker		Pharynx		Tribocytic organ	
	length	width	length	width	length	width	length	width
Dumbo, 2014 Varzea lakes/Am *Acestrorhynchus falcirostris* N = 34	1665 (1330-2466)	-	59 (28-77)	57 (35-77)	49 (21-84)	39 (20-90)	309 (112-420)	248 (150-396)
Pereira, 2016 Amazon basin *Cichla monoculus* N = 07	1438 (1178-1956)	_	63 (53-66)	67 (52-74)	61 (56-64)	50 (45-54)	335 (279-460)	194 (147-256)
Pereira, 2016 Amazon basin *Hoplias malabaricus* N = 01	1253	_	53	61	42	49	218	181
Albuquerque et al., 2017 Lake Catalão *Plagioscion squamosissimus* N = 15	1783 (148-2024)	662 (589-782)	60 (44-72)	70 (48-82)	63 (47-79)	57 (41-69)	345 (230-425)	181 (122-238)
Morais and Malta, 2015 Amazonian lakes *Pygocentrus nattereri* N *= (02)*	2050 (1370-2880)	-	66 (43-110)	-	73 (59-92)		-	280 (260-370)
Present study Igarapé, Balbina-Am *Colossoma macropomum* N = 13	1619 (1274-1844)	662 (554-902)	81 (66-89)	68 (64-77)	79 (79-80)	65 (63-78)	348 (312-392)	177 (135-227)

**Figure 2 -:**
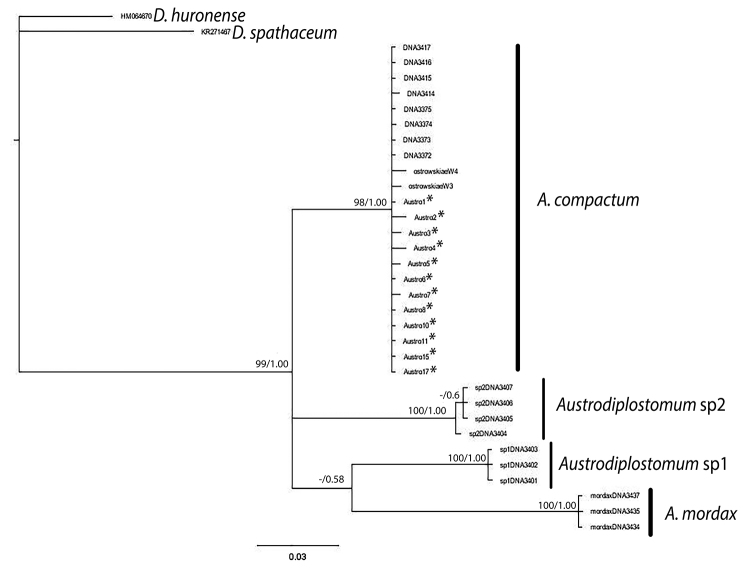
Bayesian inference (BI) tree inferred with partial sequences of COI mtDNA. The tree was reconstructed using 12 news *A. compactum* sequences, marked by asterisk (present study). In the analysis are observed 4 main subclades for *Austrodiplostomum*. Bootstrap support and posterior probability values are given next to each branch. The bar indicates the expected number of substitutions per site

Molecular analyses indicated that sequences obtained from the eyes of tambaqui belong to the species *A. compactum* (Figure 2). The *Austrodiplostomum* genus was considered by García-Varela et al. (2016) to contain three species: *A. mordax, A. compactum* and *A. ostrowskiae*. However, studies by Sereno-Uribe et al. (2019) of the three forms of *Austrodiplostomum* concluded that *A. ostrowskiae* was synonymous with *A. compactum*. 

The records collected by the current study represent the first for the State of Amazonas of an outbreak of diplostomids in farmed fish stock. It is also the first report of the genus *Austrodiplostomum* in the eyes (aqueous humor) of tambaqui, *C. macropomum,* the most important species for commercial fish farming in northern Brazil. An infective triad formed by mollusks, piscivorous birds (Cormorant) and tambaqui fry was recorded at the Fry Production Technology and Training Center, Vila Balbina/Presidente Figueiredo in the Metropolitan Region of Manaus, Central Amazon, Brazil. Parasitic helminths are important agents in the etiology of different fish diseases and can harm their hosts in a variety of ways (Eiras, 1994). The literature on the occurrence of *A. compactum* indicates a marked preference for the eye region (aqueous humor/retina/crystalline) (Santos et al., 2002; Martins et al., 2002; Abdallah et al., 2005; Azevedo et al., 2006; Bachmann et al., 2007; Yamada et al., 2008; Santos *et al*., 2012). However, metacercariae of this species may also occur in other organs, such as the brain and gills (Brasil-Sato and Santos, 2005; Machado et al., 2005). The presence of metacercaria in the eyes of farmed fish can cause mild optical damage, or have more severe impacts such as cataracts, blood vessel obstruction, retinal detachment and blindness (Monteiro et al., 2016; Lima et al., 2019).

In the current study, corneal opacity was observed in 30% of the sampled individuals. However, it is unlikely that damage percentage could have been much higher because the parasitic intensity was low, ranging from one to eight larvae per analysed eye. There was also a certain lethargy on the part of the fish at the time of capture in the tanks. This aspect of parasitism makes the fish more easily caught by cormorants. 

The registration and identification of a parasite’s biodiversity in a given locality are the first steps in carrying out control measures. It is very important to collect these data to guide technical professionals in the diagnosis of diseases. In this case, the lack of knowledge about the occurrence of pathogens and the absence of sanitary measures in the transport of fingerlings between properties can trigger dissemination processes that later are difficult to control. Therefore, the results of this study indicate that diplostomid samples collected in the vitreous humor of *C. macropomum* belong to the species *A. compactum*. These data put an alert because they include the State of Amazonas as an occurrence region of this species in addition to recording for the first time the existence of diplostomids in Amazon pisciculture. This discovery may be important because many aquaculture properties located in the Presidente Figueiredo region present conditions for the development of this parasite life cycle. In addition, many fish farms in the surroundings are supplied with fingerlings from Balbina Station.
